# On-Target Side Effects of Targeted Therapeutics of Cancer

**DOI:** 10.3389/pore.2022.1610694

**Published:** 2022-09-23

**Authors:** József Tímár, Andrea Uhlyarik

**Affiliations:** ^1^ Departments of Pathology, Forensic and Insurance Medicine, Semmelweis University, Budapest, Hungary; ^2^ Internal Medicine and Oncology, Semmelweis University, Budapest, Hungary

**Keywords:** immunotherapy, target therapy, bone therapy, angiogenesis inhibitor, side effect, pathomechanism

## Abstract

The concept of precision medicine is based on the identification of hallmarks of cancer to exploit them as drug targets. The basic idea was that in this way the therapeutic modalities will be more effective and the side effects will be less. Since the majority of these novel modalities are not specific for a cancer-related biological process or a cancer-specific (mutant) target protein, it is not a surprise that we had to learn new type of side effects, because these therapeutics also affect physiological or pathological processes. Even more, in cases of some of these novel therapies we were able to discover new molecular mechanisms of physiological and pathological processes. Identification of the on-target side effects of targeted drugs can help to prevent the development of them or better manage the patients when emerge during cancer therapy.

## Introduction

Precision medicine emerged on the basis of genetic characterization of various cancers, establishing molecular classifications paving the way for the development of various target therapies. Major aim was to design more tumor-specific therapies to spare normal tissues. Today the majority of precision medicine drugs are not specific for mutant targets of the cancer cells since they are active on wild type targets as well, opening the way for the development of the so-called on-target side effects. Although, some of the novel targeted drugs are selective for mutant targets, they can affect wild type ones as well. This is especially true for angiogenesis inhibitors, bone metastasis inhibitors or the new immune checkpoint inhibitors. Herein we summarize the on-target side effects of the target therapies and the underlying pathological or molecular mechanisms behind them, since recognition of those mechanisms can prevent side effect development or can help to manage of them.

## Angiogenesis inhibitors

It is one of the hallmark of cancer that it can provide its own vascularization beyond the growth of one mm^3^ (1). Cancer tissue vascularization mechanism has various forms but most frequently involves neoangiogenesis. Major trigger and regulator of this process is the cancer hypoxia (either true or mimicked) directed by HIF transcription factors activities leading to production of angiogenic cytokines, mainly VEGFA (1). When the basic pathomechanism was revealed new therapeutic approaches have been developed to neutralize VEGF or inhibit the tyrosine kinase activity of its receptors VEGFR1/2. Interestingly, while these novel anti-angiogenic agents were able to fulfill the expectations by inhibiting tumor induced neoangiogenesis in preclinical models, but they were not able to do it in cancer patients, rather their effect was mainly the “normalization” of the tumor’s aberrant vasculature (2) Meanwhile, novel unforeseen side effects emerged upon such therapies: bleeding, wound healing disturbances, hypertension and the question immediately raised if those side effects are on-target ones?

### Bleeding and Thromboembolic Event

Production and the physiological distribution of VEGF was not known before. When the biodistribution of anti-VEGF antibodies was analysed in human, it was noted that it stays in the circulation for hours and it was found to be incorporated into platelets. These studies identified platelets as the largest VEGF depo in the body, which fact explains why anti-VEGF antibody therapies disturb platelet functions and cause bleeding and thrombotic events (3).

Other characteristic side effect of the anti-angiogenic agents is arterial thrombotic events, the pathomechnism of which is different form the above mentioned one. These agents has a strong effect on normal microcapillary systems and have an endothelial- and a pericyte-dependent component leading to the loss of vasodilatation potential. Furthermore, angiogenesis inhibitors, especially the so called multikinase ones, are characterized by depletion of pericytes in microcapillaries, most probably due to the inhibition of PDGFR activities. These combined effects on endothelial cells and pericytes, increase the risk of rupture of atherosclerotic plaques leading to thrombotic complications upon therapies (4).

### Wound Healing Disturbances

**FIGURE 1:**
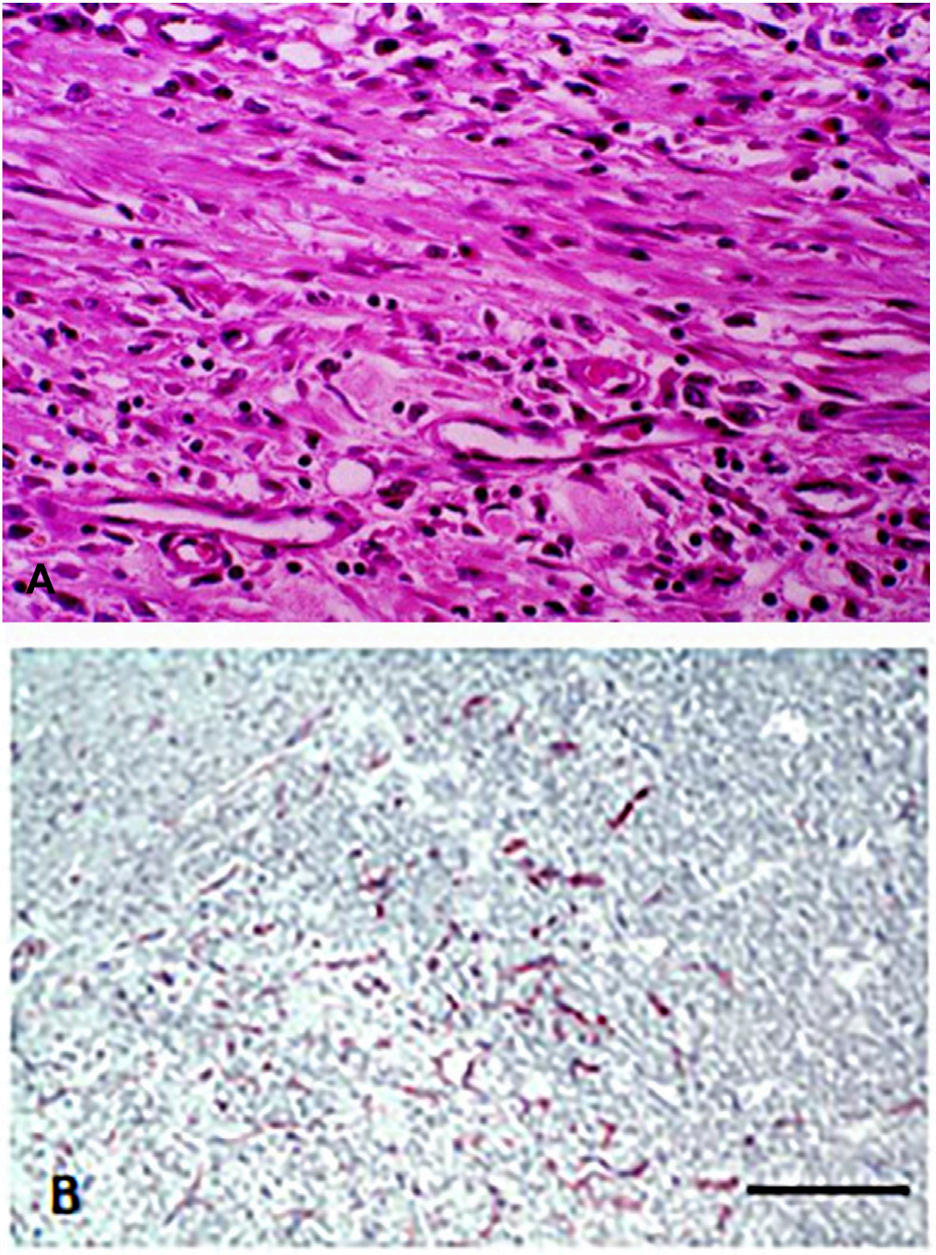
Capillary proliferation in chronic inflammation. **(A)** Proliferating fibroblasts and capillaries in granulation tissue. HE staining. Bar: 100 μm. **(B)** Immunohistochemical labeling of endothelial cells in granulation tissue. Note that beside small capillaries, individual endothelial cells are also present. CD34 immunolabeling. Bar: 200 μm. With permission of Hungarian Society of Clinical Oncology (5).

Mechanical injuries to various tissues, especially surgical wounds are healed by formation of granulation tissues. Which is composed of proliferating capillaries, fibroblasts and chronic inflammatory cells (Figure 1.) and the wound is replaced by novel vascularized connective tissue. Although angiogenesis inhibitors are unable to inhibit tumor-induced neoangiogenesis, they are effective inhibitors of the sprouting type capillarogenesis, which will lead to wound healing disturbances in cancer patients (1).

### Hypertension

Hypertension is a classical side effect of angiogenesis inhibitors, but the anti-VEGF agents are better from this aspect than the VEGFR inhibitors. In experimental models it was shown that anti-VEGF agents induce acute hypotension which can be treated by NOS inhibitors. On the other hand, studies revealed that the developing hypertension in treated cancer patients is due to the decreased capillary densitiy of the renal medulla, again providing evidence for the characteristic effects on microcapillaries (6).

### HIF2a Inhibitor: Anemia

Recently a novel class of anti-angiogenic agents entered into clinical practice: the HIF2a inhibitor, belzutifan. This novel drug is designed to block HIF2a activity and prevents the induction of specific HIF-regulated genes such as VEGFA, EPO, GLUT1 but leaves unaffected CA9 or LDHA (regulated by HIF1a) (7). This drug obtained FDA approval in VHL-syndrome patients with cRCC, CNS-hemangioblastoma or pancreatic NET (8). Belzutifan is efficiently inhibits VHL-mutated tumor growth, but blocking of EPO expression by belzutifan results in treated patients decreased RBC count, HgB levels and consequent systemic hypoxia (9). Due to the mechanism of action of this drug, the use of erythropoetin stimulating agents is contraindicated to treat these side effects.

## Bone Metastasis Inhibitors: Bisphosphonates and Anti-RANKL Antibodies

**FIGURE 2:**
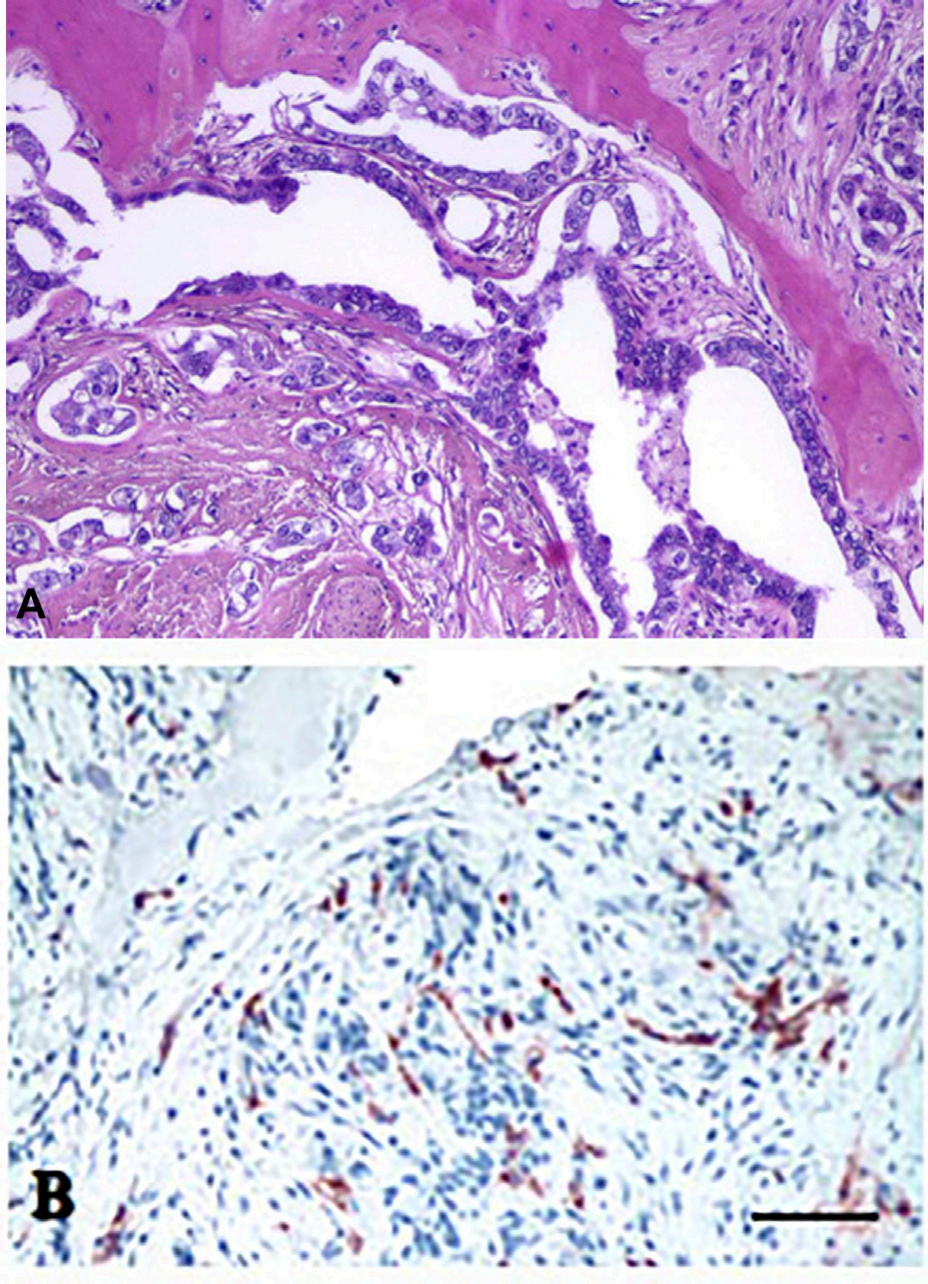
Histology of bone metastasis of breast cancer. **(A)** HE staining. **(B)** Osteoclast labeling of bone metastasis. Tartarate-resistant acid phosphatase reaction. Bars: 200 μm. With permission of Hungarian Society of Clinical Oncology (5).

**FIGURE 3:**
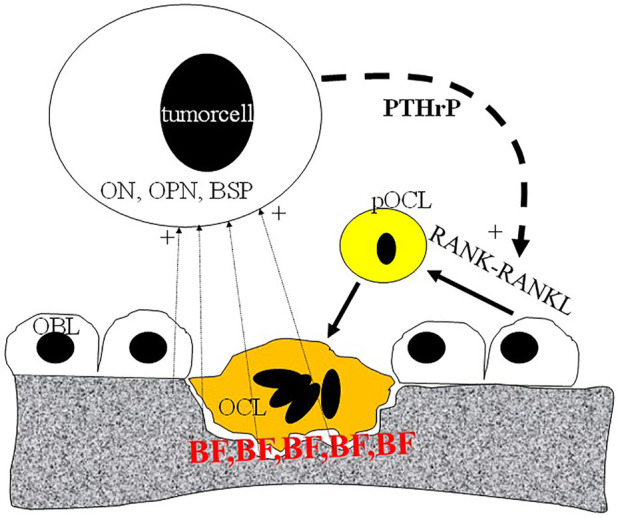
Schematic representation of metastatic tumor cell-bone tissue interaction. Tumor cells produce PTHrP (parathromone-like protein) as well as bone-specific matrix components osteopontin (OPN), osteonectin (ON) and bone sialoprotein (BSP). PTHrP stimulate osteoblasts (OBL) to differentiate into pre-osteoclast (pOCL) mediated by the RANKL/RANK receptor system. Ultimately activated osteoclasts degrade the mineralized bone tissue which process is blocked by bisphosphonates (BP) deposited into the bone tissue.

Development of bone metastasis of various cancers has a common pathomechanism: cooperative interaction of cancer cells with the bone microenvironment as well as with osteoblasts and more importantly osteoclasts (Figure 2). Cancer cells which are capable for bone metastassis formation are producing PTHrP, which promotes osteoclast differentiation and activity through the RANK/RANKL receptor system (Figure 3). Based on this mechanism, two types of therapies have been developed, and both are targeting osteoclasts: anti-RANKL antibodies and bisphosphonates (BF). The latest drug types have been designed to target osteoclasts, since they accumulate preferentially in mineralized bones. Bisphosphonates form ATP-conjugates and this way they block activation of osteoclast in bone resorption and induce their apoptosis. The second generation bisphosphonates (amino-BF) have another mechanism of action, they are effective prenylation inhibitors by this secondary activity they activate macrophages and T cells resulting in the increased TNFa and IL-6 levels (10). These differences in mechanism of action are important to understand the development of side effects upon their application (11).

Both bisphosphonates and anti-RANKL antibodies induce therapy resistant osteonecrosis (12, 13). The cause of that particular side effect is the inhibition of osteoclast activities necessary for bone remodeling and regeneration. Such processes are very active in case of acute periodontitis which can turn into massive osteonecrosis upon bone metastasis inhibitor treatment (14).

Use of aminobisphosphonates can result in the development of another type of side-effect: fever and acute phase reaction. Caused by activation of macrophages and release of inflammatory cytokines. On the other hand, in case of anti-RANKL antibody therapies infections may emerge due to the fact that these antibodies accumulate in lymph nodes as well, where their inhibitory effect on macrophages may result in the inhibition of acute inflammatory responses (9, 10). Today, bone metastasis therapies can be combined with anti-angiogenic therapeutics which further increase the risk of development of osteonecrosis, since bone remodeling and regeneration in case of periodontitis requires also formation of novel capillaries (12, 13, 14).

## Immune Checkpoint Inhibitors

Discovery of immune checkpoints revolutionized the way we think about immune regulation. In case of adaptive immunity the regulatory phase seems to be equally important as compared to stimulatory phase, since that ensures the development of immune tolerance. This process is regulated by immune checkpoints, PD1, TIGIT, TIM-3, CD-200R1, BTLA, VISTA, LAG-3 and CTLA-4 expressed by activated T cells while their ligands are expressed mostly by dendritic cells and T cells, but some of them can be expressed by peripheral non-immune cells as well (PDL1/2, CD-200 or PSGL1). The role of CTLA-4 is to block antigen presentation by inhibiting the activation of CD28 and preventing IL-2 production. CTLA-4 is also expressed by Treg cells maintaining long lasting immunotolerance. Expression of PD1 is relatively broader, since beside T cells, it is expressed by B cells, NK cells and macrophages (15). Knocking out PD1 in animal models results in the development of lupus-like systemic autoimmune diseases, On the other hand, knocking out CTLA-4 in animals, leads to more fatal autoimmune diseases as compared to PD-1 knockouts (16, 17). Since immune checkpoints play a crucial role in anti-tumoral immune responses, the introduction of immune checkpoint inhibitors revolutionized cancer management, but resulted in the emergence of a new class of side effects, autoimmune diseases.

### Anti-CTLA4 Antibody Therapy

Administration of Ipilimumab (anti-CTLA-4 antibody) to cancer patients induces dose dependently immune-related adverse events (irAE), at higher doses the frequency of grade >3 events is 37% while at lower doses 18%, developing after 2–3 months of therapy. The most frequent irAE is colitis, but dermatitis, hepatitis, endocrinopathies (thyreoiditis, hypophysitis) are also prevalent. Interestingly, at lower rate myasthenia gravis, uveitis, ganglionopathies or even autoimmune thrombocytopenia can also occur (18).

### Anti-PD-1 Antibody Therapies

The serious irAE incidence of anti-PD1 therapies is lower as compared to anti-CTLA-4 (grade >3irAE <10%), but unfortunately they can occur later, can be long lasting, and more difficult to treat. Forms of these autoimmune diseases are similar to anti-CTLA-4 therapy, except arthritis and pneumonitis (19, 20).

### Anti-PDL1 Antibody Therapies

The irAE incidence of these therapies is lower as compared to anti-PD1, since this type of treatment does not affect the PDL2/PD1 axis and for example the incidence of pneumonitis is half as compared to anti-PD1 therapies (21, 22, 23).

### Anti-CTLA4 and anti-PD1 Combination Therapies

Although the anti-tumor efficacy of this combination immunotherapy is much higher, it also increase the incidence rate of irAE which are also more serious as compared to monotherapies. The most frequent forms are colitis, dermatitis, endocrinopathies and hepatitis (24, 25). In NSCLC patients in the combination arm myocarditis, pneumonitis and renal insufficiency predominated (26, 27, 28). A summary of irAEs of immune checkpoint inhibitors is presented on Table 1.

**TABLE 1:** Autoimmune side effects of the immuncheckpoint inhibitors. (see refs. (17-27)).

	Anti-PD1/anti-PDL1 antibodies	Anti-CTLA4 antibody	Combination
Dermatitis	+	+	+
Colitis	+	+	+
Pneumonitis	+		+
Endocrinopathy	+ (thyreoiditis)	++ (hypophysitis)	++
Hepatitis	+	+	++
Myocarditis			+
Autoimmune events of the nervous system		+	+
Arthritis	+		+

#### Dermatitis

This is one of the most frequent irAE in case of checkpoint inhibitor therapies and occurs as lichenoid or spongiotic form. Extreme forms are involve more than 30% of the body surface. The autoimmune inflammation takes place at the interface of the epidermis-dermis and involves the accessory organs of the dermis (hair bulb and sweat glands). The incidence of cutaneous inflammations in psoriatic patients is more frequent but the treatment also activate the original disease. Frequently the autoimmune inflammation also involves mucosal surfaces as well (18, 19, 20, 21, 22, 23, 24, 25, 26, 27, 28).

#### Colitis

This inflammation type is also very frequent in immune checkpoint inhibitor therapies, especially in case of anti-CTLA-4 antibody administration. It appears as ulcerative colitis with epithelial damage and crypt abscess infiltrated by mixed immune cells (leukocytes and lymphocytes). The severity of the inflammation must be determined from colonic biopsy specimen. In case of the predominant ulcerations, massive anti-inflammatory treatment can be necessary. The key factor behind this induced autoimmune disease is the depletion of the mucosal Treg cells, which is more prevalent in patients with CTLA-4 polymorphism (Y60C). Interestingly, the pathomechanism of the anti-PD1/PDL1 antibody treatment induced enteritis is different, since all inflammatory cells overexpress PD1. Furthermore, the composition of the patient’s microbiome is also an important factor, since the incidence of colitis is higher in those patients where their microbiome is lacking bacteria which are poliamine transport- or B vitamin biosynthesis negatives (18, 19, 20, 21, 22, 23, 24, 25, 26, 27, 28).

#### Pneumonitis

**FIGURE 4:**
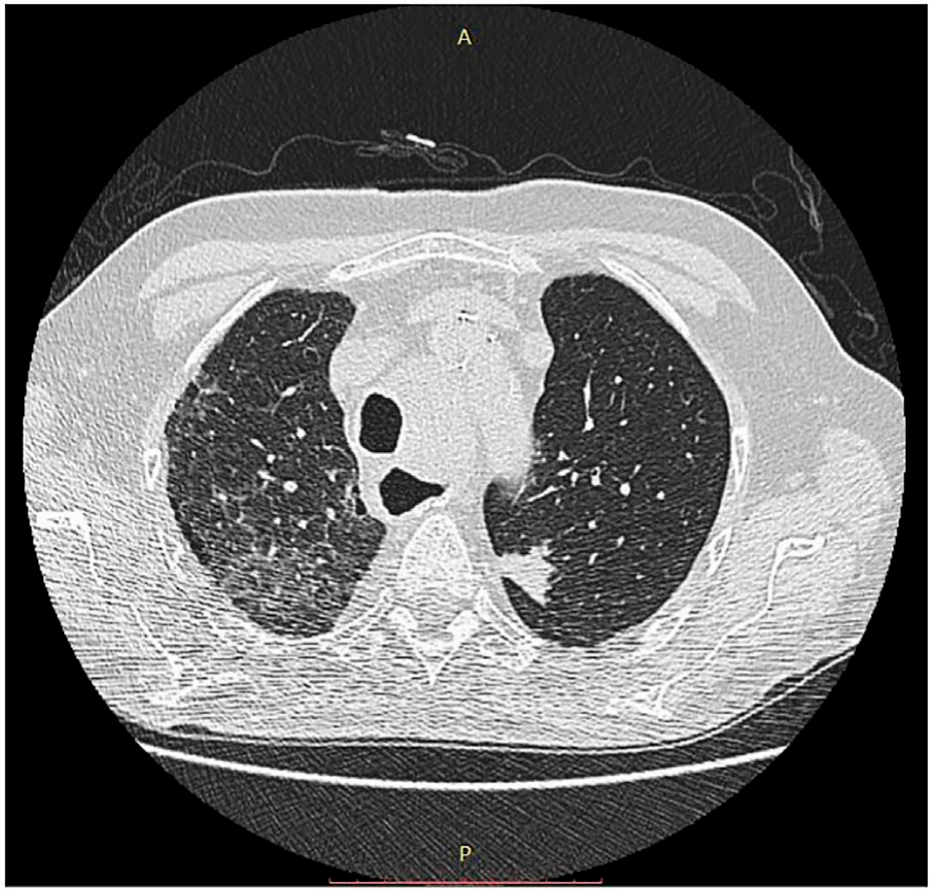
CT image of pneumonitis of pembrolizumab-treated cancer patient. With permission of Hungarian Society of Clinical Oncology (5).

Autoimmune disease of the lung as irAE is the characteristics of the anti-PD1/PDL1 therapies and is more frequent in the COPD patients. The pathological spectrum is broad from ARDS and interstitial pneumonitis to sarcoidosis-like forms. Among fatal irAE pneumonitis is the leading cause, therefore early diagnosis is very important. Beside COPD other predisposing factors are smoking, and previous chemo- or radiotherapy. The bronchioalveolar lavage used to be lymphocyte-rich where the physiological ratio of CD4/CD8 is changed significantly (18, 19, 20, 21, 22, 23, 24, 25, 26, 27, 28) (Figure 4).

#### Endocrinopathies

Hypophysitis is a novel autoimmune disease specific for immune checkpoint inhibitor therapies, especially of the anti-CTLA-4 one. This is an acute inflammation of the anterior lobe of the hypophysis which may lead to compression of the chiasma opticum. Peripheral consequences of the hypophysitis can be adrenal gland insufficiency, thyroid dysfunction or gonadal insufficiencies. In this autoimmune complication one can detect circulating autoantibodies against TSH-, FSH- or ACTH-producing cells. It is also important that the TSH- and prolactin-producing cells spontaneously express CTLA-4. It is also important, that the inflammation takes place in form of type-II hypersensitivity reaction. It is also interesting that in case of anti-PD1/PDL1 therapies there is another target organ which the thyroid. These therapies initially inducing hyperfunction which later turn into hypothyreosis (18, 19, 20, 21, 22, 23, 24, 25, 26, 27, 28).

#### Hepatitis

Administration of immuncheckpoint inhibitors, especially in combination, frequently results in hepatitis diagnosed by increased (>3x) transaminase levels, when biopsy is indicated. Histopathologically it is characterized by panlobular inflammation which is accompanied by focal or confluent hepatocyte necrosis. It can appear as lobular granulomatous inflammation, when histiocytes accumulate around lipid vacuoles and fibrin (18, 19, 20, 21, 22, 23, 24, 25, 26, 27, 28).

#### Myocarditis, Myositis

This is a rare form of irAE in case of immunecheckpoint inhibitor therapies but among fatal cases it is relatively more frequent. Histologically myocarditis is characterized by an inflammatory exudate rich in T cells, but lacking immuncomplex deposition. In a small proportion of treated patients myositis may also occur, which can lead to myasthenia gravis and necrosis of muscles. Histologically, there is a peri- and endomysial mixed inflammatory exudate of T-, B-cells and macrophages, however, anti-AchR or myositis-autoantibodies are missing (18, 19, 20, 21, 22, 23, 24, 25, 26, 27, 28).

#### Neurological Autoimmune Diseases

Administration of checkpoint inhibitors may induce autoimmune reactions in the CNS in form of aseptic meningitis or encephalitis. Since the symptoms can be indispensable from CNS metastasis the diagnosis is very important based on the examination of the liquor or serum since in this irAE autoantibodies can be detected against NMDA receptor, contactin-associated protein-2 or Hu protein. Autoimmune inflammation can also be induced in the peripheral nerves affecting the neuromuscular junction or the nerves itself leading to demyelinization (18, 19, 20, 21, 22, 23, 24, 25, 26, 27, 28).

### Autoimmune Adverse Events as Predictive Markers of the Efficacy of Checkpoint Inhibitor Therapies

Since immuncheckpoint inhibitors are not cancer-specific or selective treatments, they enhance the activity of the entire adaptive immune system resulting in the above mentioned autoimmune adverse events. That was the reason, why it was proposed that the autoimmune adverse events can be used as positive predictive marker for anti-cancer efficacies of these therapies. Such studies mostly done in case of melanoma, which demonstrated that emergence of early immunological adverse events (after 12 weeks) significantly associated with overall survival and it has an independent predictive power (29, 30). A meta-analysis of ICI-treated cancer patients indicated that the OS and PFS of those patients who has irAE is significantly longer (31). It is of note, that not all type of irAE has such a predictive value, but dermatitis, endocrinopathy and colitis, exclusively. In case of melanoma patients it was an early observation that upon ICI treatment vitiligo may appear in the dermis and this clinical feature may be used also as a positive predictive marker (32). This side effect is based on the fact that melanocytes and melanoma cells share several common protein markers, mostly melanosome-associated ones, therefore the antitumoral immune response can be also anti-melanocyte one.

## Oncogene Targeted Therapies

### Anti-HER-2 Antibodies and HER-2 Inhibitors

**FIGURE 5:**
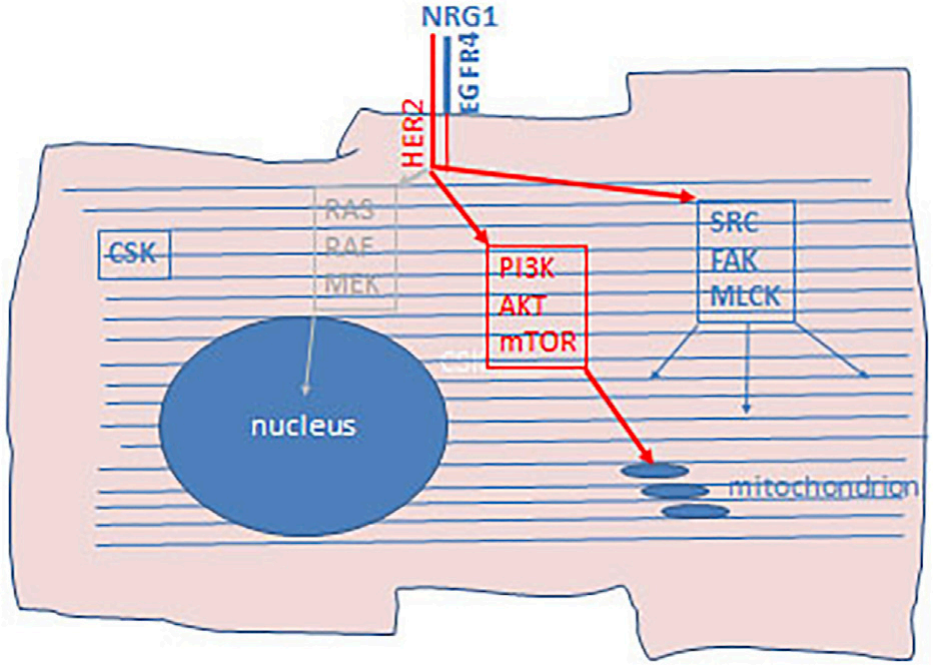
Schematic presentation of cardiotixicity pathway of HER-2 inhibitors. Cardiomyocytes express HER-2/EGFR4 aspecially in hypoxic condition as well as neuregulin-1 (NRG1). In adult differentiated cardiocytes the RAS-RAF-MEK mitotic arm of this signaling pathway does not operational. Upon HER-2 inhibition PI3K/AKT/mTOR arm of the signaling pathway is blocked which activate mitochondrial apoptotic pathway. Furthermore, since the SRC/FAK/MLCK arm of this signaling pathway is also blocked, regeneration of the actomyosin complex is also inhibited. CSK, cytoskeleton.

Identification of the molecular subclass of breast cancer, the HER-2 amplified form, initiated a revolutionary change in the treatment of this cancer, originally using anti-HER-2 antibodies, later complementing it with small molecular TK inhibitors (33). However, it was early discovered that the administration of these therapies has a unique side effect, the cardiotoxicity due to decreased left ventricle ejection fraction. Furthermore, it was clear that the risk of cardiotoxicity dramatically increases when this type of target therapy is combined with the commonly used chemotherapy, antracyclins. Studies revealed that HER-2 and EGFR4 receptors play a crucial role in cardiomyocytes (34). HER-2 receptor is a ligand-less kinase receptor, whereas EGFR4 has ligand which is NRG1 in case of myocardium. The NRG1-EGFR-4/HER-2 receptor complex and the associated signaling pathway plays a key role during embryonal development of the heart. In adults, this receptor complex is involved in the everyday function of the heart muscle, in the accomodative hypertrophy and muscle cell survival upon various form of stress. In other cell types activation of HER-2 results in the activation of the mitotic signaling pathway (RAS-MEK-ERK), but not in cardiomyocytes. In differentiated cardiomyocytes the HER-2/EGFR4 signaling pathway activates the lipidkinase (PI3K/AKT) and the SRC/FAK arm of that pathway. The target of the lipidkinase pathway activation is the NOS-system, to decrease the production of free radicals, stimulate the β-adrenergic activity and block apoptotic pathways. Stimulation of the SRC/FAK pathway is responsible for actomyosin function by the activation of MLCK (Figure 5). Blocking of HER-2 decreases the NOS activity thereby increasing ROS levels leading to disturbed mitochondrial function in cardiomyocytes. Furthermore, HER-2 blocking and the inhibited PI3K/AKT activity induces the intrinsic mitochondrion-related apoptotic pathway by increasing BCL2 and decreasing BCL-XL levels. Histologically, swelling of the mitochondria in cardiomycytes is a hallmark of this effect. On the other hand, decreased MLCK activity promote disintegration of the actomyosin complex, also recognizable by histology (33, 34).

**FIGURE 6:**
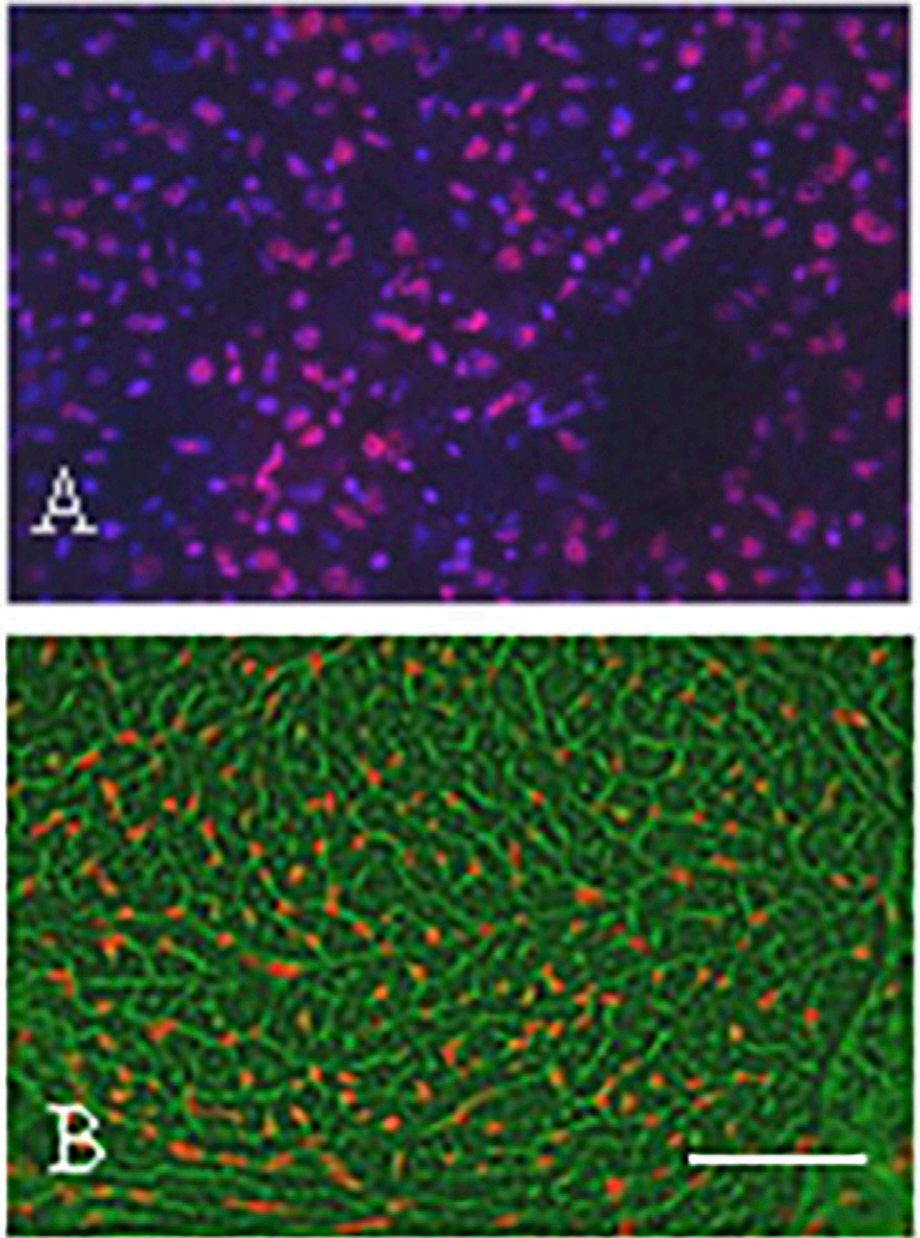
Demonstration of the presence of epirubicin (red autofluorescence) in SCID mice after 5 min of i.v. administration using fluorescence microscopy of frozen sections. **(A)** Subcutaneously growing human breast cancer tumor tissue. Note the red fluorescence of the tumor cell nuclei. Bar: 200 μm. **(B)** Cardiac muscle tissue. Note the red fluorescence in the nuclei of the cardiomyocytes. Bar: 400 μm. With permission of Hungarian Society of Clinical Oncology (5).

As mentioned before, the risk of cardial side effects is higher when anti-HER-2 therapies are combined with antracyclin chemotherapy of breast cancer. One of the main cause of the cardiotoxicity of antracyclins is the fact that this drug rapidly accumulate in the nuclei of cardiomyocytes (since this is a DNA-binding molecule) of the well-perfused heart after i.v. administration (Figure 6). Antracyclins induce vacuolar degeneration and cell necrosis, irreversible damages in the heart muscle, while the effects of the anti-HER2 therapies is reversible in heart muscle, therefore after suspension of the administration of the targeted drugs cardiac functions usually improve. The antracyclin-induced cardiotoxicity (cell stress) activates the NRG1/EGFR4/HER2 receptor complex, which improves the survival of the cardiomyocytes. However, when the two drugs are combined, the adaptive potential of the heart muscle decreases leading to irreversible myocyte damages (apoptosis and necrosis) (35).

### EGFR Inhibitors

**FIGURE 7:**
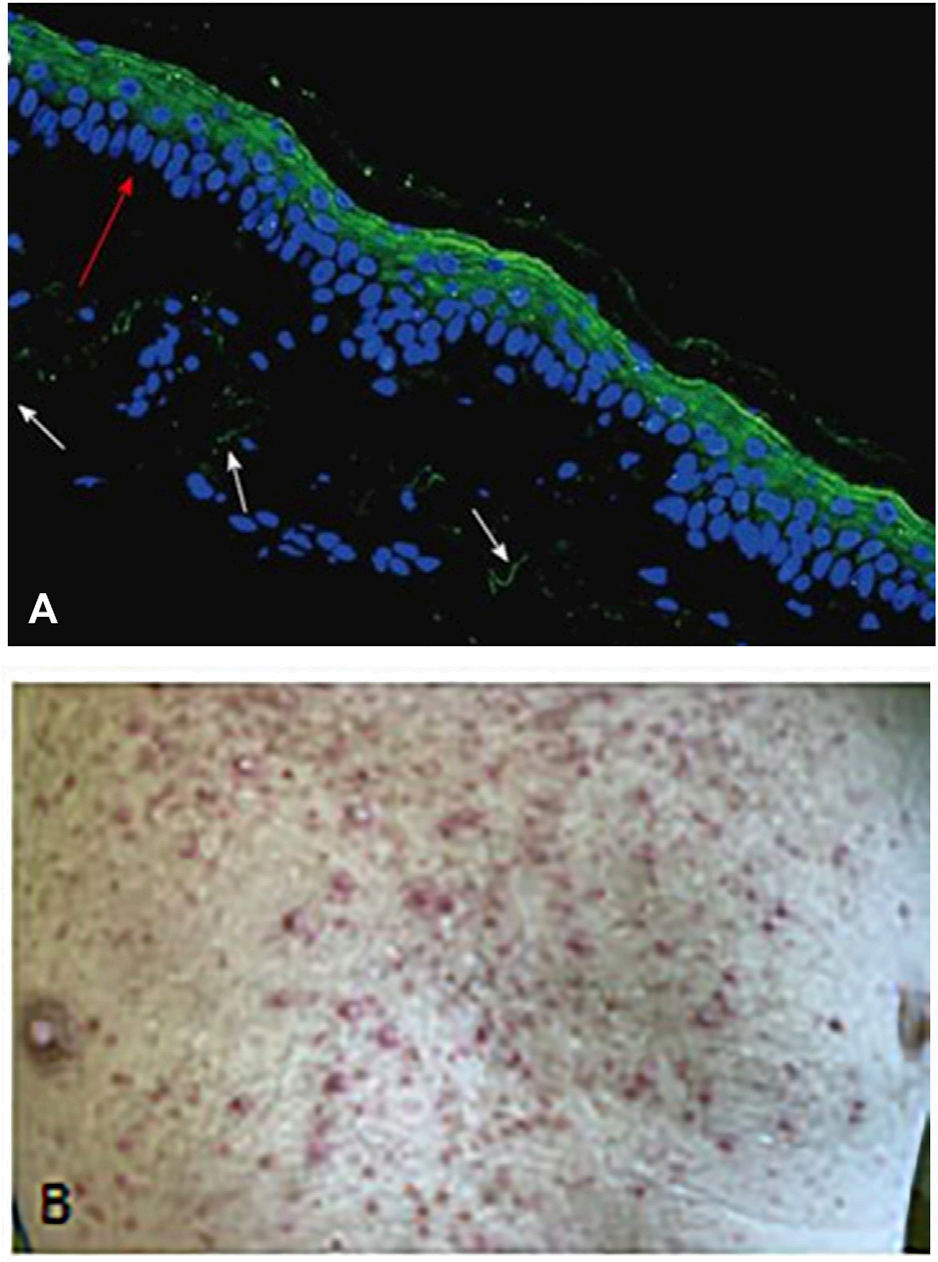
Demonstration of skin rash induced by anti-EGFR treatment (Cetuximab) of colorectal cancer patient. **(A)** EGFR1 protein in dermal epithelium of frozen section of the human skin using anti-EGFR antibody (cytoplasmic domain) and FITC labelled secondary anti-mouse antibody. Note the intense labeling of the epithelial cells (green fluorescence). Bar: 100 μm. **(B)** Macrosopic picure of skin rash. With permission of Hungarian Society of Clinical Oncology (5).

EGF is the most important regeneratory ligand of the skin epithelia, but it is also involved in maintenance of the integrity of the epidermis and the function of the accessory glands. EGF acts through the EGFR1 receptor, which is therefore constitutively expressed by the epithelial cells (Figure 7). Anti-EGFR antibody- and EGFR-TK inhibitor therapies are all characterized by inducing dry skin, acneiform rashes, and decrease in the defense function leading to infections (36). One of the consequence of this is the bacterial folliculitis. Since the growth of nails is also governed by EGF/EGFR1, it is not much a surprise that these therapies induce paronychia, inflammation of the periungual tissues. Hair growth is also regulated by EGF/EGFR1, accordingly, the anti-EGFR therapies has significant side effects. Since EGFR1 is the initiator of the growth phase (anagen) of hair, significant disturbances occur upon therapeutic inhibition leading to folliculitis and follicular necrosis as well as alopecia. Although these side effects used to be low grade, but can lead to the dose-decrease of these drugs. The first generation EGFR TK inhibitors are not mutation selective unlike the second and third generation ones, where the incidence of these skin toxicities is much lower (37).

### BRAF Inhibitors

**FIGURE 8:**
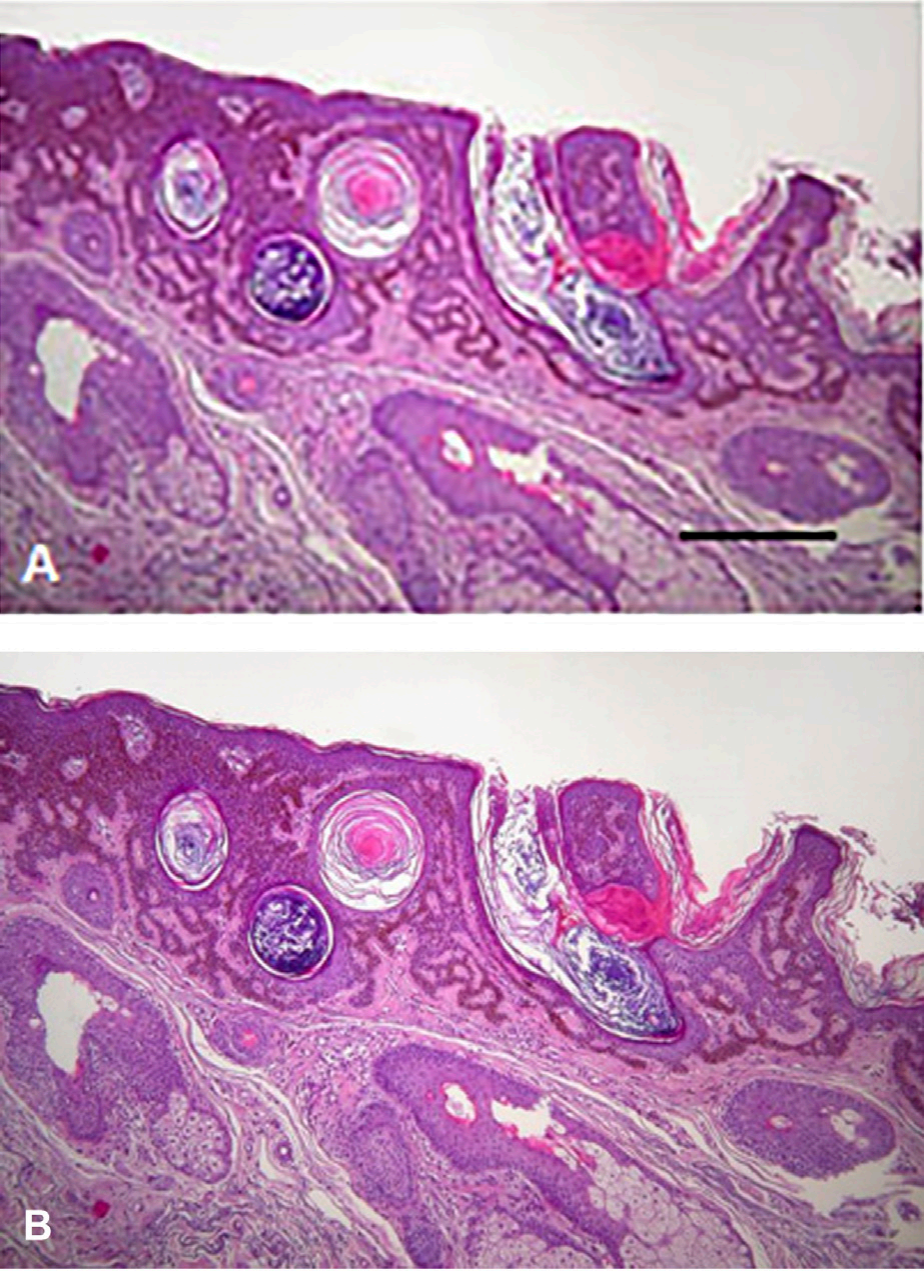
Histology of skin tumors induced by BRAF inhibitor therapy of melanoma patients. **(A)** Keratoacantoma. Bar: 400 μm. **(B)** Squamous carcinoma. HE staining. Bar: 200 μm. With permission of Hungarian Society of Clinical Oncology (5).

BRAF mutant tumors (melanoma, lung adenocarcinoma anaplastic thyroid- or colon cancer) can be treated by BRAF inhibitors (38). Although these tumors used to respond well to such a target therapy, this type of drugs were the first where upon administration of a target therapy for one cancer type induced the development of another(39, 40). In melanoma patients treated with BRAF inhibitors a characteristic side effect can be observed with high frequency, development of benign epithelial tumors (keratoacantoma, papilloma) or malignant ones (squamous cancer) (Figure 8) as well as new nevi or induced novel melanomas. However, these tumors can easily be detected and treated appropriately. Unfortunately, BRAF inhibitors can induce other cancer types as well, such as head and neck-, colorectal cancer or glioblastoma.

**FIGURE 9:**
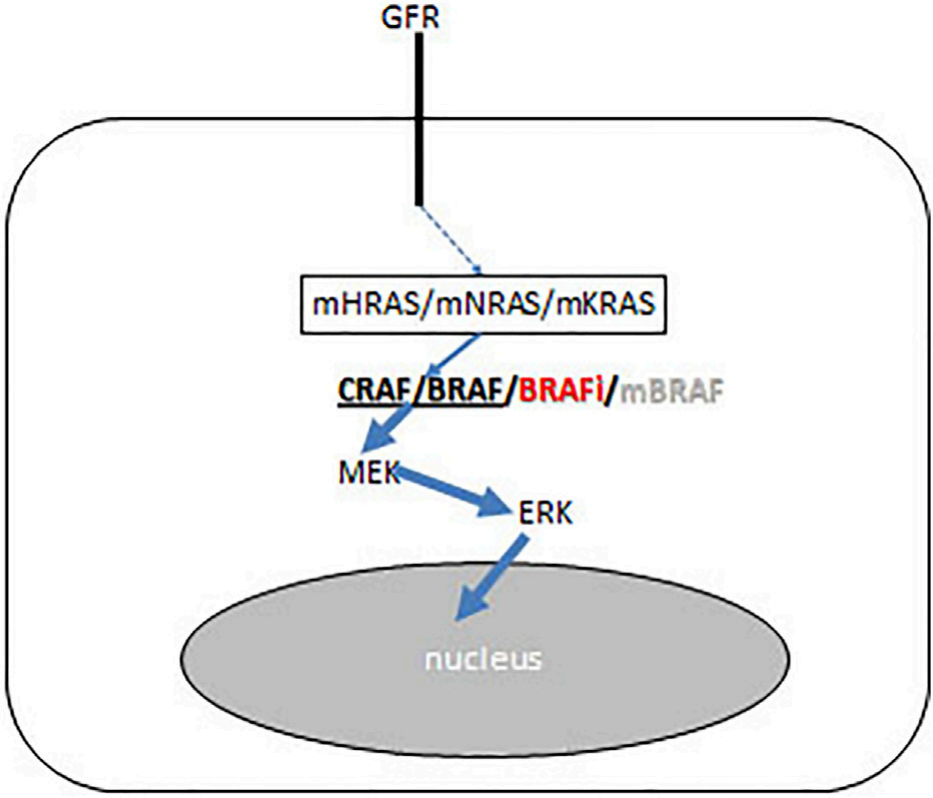
Schematic presentation of BRAF inhibitor effect on the growth factor receptor (GFR) signaling pathways of precursor cancer cells where carcinogenesis iniciated by RAS mutation (KRAS/NRAS/HRAS). BRAF inhibitor induces CRAF/BRAF homo- and heterodimerisation and activates the RAF-MEK ERK mitogenic signaling pathway. Arrow thickness represents signal intensities.

BRAF inhibitors designed as mutant-selective small molecular inhibitors but they have an inhibitory effect on the wild type protein as well. In normal cells BRAF inhibitors induce the activation of the MAPK pathway, due to the induced homo-or heterodimer formation of wild type C-RAF or BRAF. Today BRAF inhibitors are used frequently in combination with MEK inhibitors, in that case of this paradoxical MAPK activation is less frequent. It is interesting, that in RAS mutant premalignant lesions this MAPK activation finalizes the carcinogenic process (Figure 9). As a result, HRAS mutant squamous- and head and neck cancers, NRAS mutant novel melanomas or myelomonocytic leukemia and KRAS mutant colorectal cancers could be developed upon BRAF inhibitor therapies (38, 39, 40).

### Multikinase Inhibitors

Advent of target therapy started with the development of ABL inhibitors for BCR-ABL fusion positive CML. The original first generation but even the later generation ABL inhibitors are still “dirty” drugs in a sense that they inhibit other kinases especially KIT. Since KIT is the master regulator of the bone marrow progenitor cells, the administration of these non-specific ABL inhibitors results in myelosupression (41, 42). Furthermore, this type of side effect characterizes also any type of KIT inhibitor. Furthermore, KIT is the master regulator of melanocyte differentiation, accordingly KIT inhibitors induce vitiligo and hair color changes (40).

Multikinase inhibitors are now widely used in several cancer types and most of them has VEGFR2 and or PDGFR inhibitory activity. Accordingly, the typical side effects of the angiogenesis inhibitors, such as bleeding and thromboembolism can be considered on-target side effects.

It is a novel class of multikinase inhibitors, the ALK inhibitors which are most effective in ALK-fusion positive lung adenocarcinoma. ALK inhibitors also have significant side effects such as pneumonitis, neurological-, cardiological-, gastrointestinal-, and urogenital toxicities but none of them was shown to be considered as on-target ones (43).

However, this cannot be applied to the new TRK inhibitors which are registered in TRK-fusion positive malignancies. TRK inhibitors have three targets, TRK-A, TRK-B and TRK-C which differ in ligands and functions. NGF is the ligand of TRK-A, BDNF and neurtrophin-4 are ligands for TRK-B while neurotrophin-3 is the ligand of TRK-C. These receptors are key regulators of the development of the nervous system. Since TRK-inhibitors are registered as tumor-agnostic therapies, it is important to consider that the development of the nervous system is ongoing in children till age two, therefore in case of TRK-fusion positive pediatric tumors the unique side effect profiles of those inhibitors must be considered. On the other hand, TRK inhibitor administration in adults can also lead to neurological toxicities and uniquely pain after cessation of therapy (44). This side effect is a typical “on-target” side effect since sensory nerves express TRK-A and NGF is an important modulator of pain. Furthermore, TRK receptors are important in various functions of ganglion cells of CNS as well, accordingly dizziness, cognitive dysfunctions or ataxia all have to be considered “on-target” side effects.

### PI3K/AKT/mTOR Inhibitors

The PI3K/AKT/mammalian target of rapamycin (PI3K/AKT/mTOR) signaling pathway plays a key role in many cellular function. The inhibitors of this signaling pathway could be divided into five subgroups, as follows: panPI3K inhibitors, dual PI3K/mTOR inhibitors, PI3K isoform-specific inhibitors, AKT inhibitors, and mTOR inhibitors. The pathomechanism of the side effects are not completely clear in all instances, but as mTOR inhibitors are immunosuppressors, such an effect must always be considered in cancer patients. Inhibition of the different PI3K isoforms could cause different side effects, but some of them should be considered as class-effect of these drugs (45). Most of the adverse events are dose dependent, but sometimes they are idiosyncratic and unpredictable (46).

#### Hyperglycemia

The PI3K/AKT/mTOR signaling pathway is one of the key effector of the insulin receptor (IR) and insulin receptor substrate proteins (IRS), and therefore it is an important regulator of the effect of insulin. The PI3K/AKT/mTOR axis regulate many targets, such as glycogen synthase kinase 3β (GSK3β) and the forkhead box protein O1 (FoxO1). These effectors increase the glycogen synthesis, and inhibit the gluconeogenesis, therefore lower the blood glucose level. It is not a surprise accordingly, that hyperglycemia is one of the more frequent adverse effect of PI3K/AKT/mTOR inhibitor treatment. The hyperglycemia, induced by PI3K inhibitors is isoform specific, mainly experienced during a PI3Kα specific treatment (45). Hyperglycemia is a major problem with PI3K inhibitors, which stimulated development of guidelines (47).

#### Hyperlipidemia

Hyperlipidemia is also a frequent side effect of mTOR inhibition therapy. The mTOR pathway is an important regulator of the lipid metabolism. The mTOR kinases form large protein complexes, the mTOR complex 1 (mTORC1) and mTOR complex 2 (mTORC2). The activated complexes enhance the accumulation of triglycerides, promote adipogenesis and lipogenesis, and inhibit catabolic processes, such as lipolysis and β-oxidation (45, 46, 47, 48).

#### Pneumonitis

**FIGURE 10:**
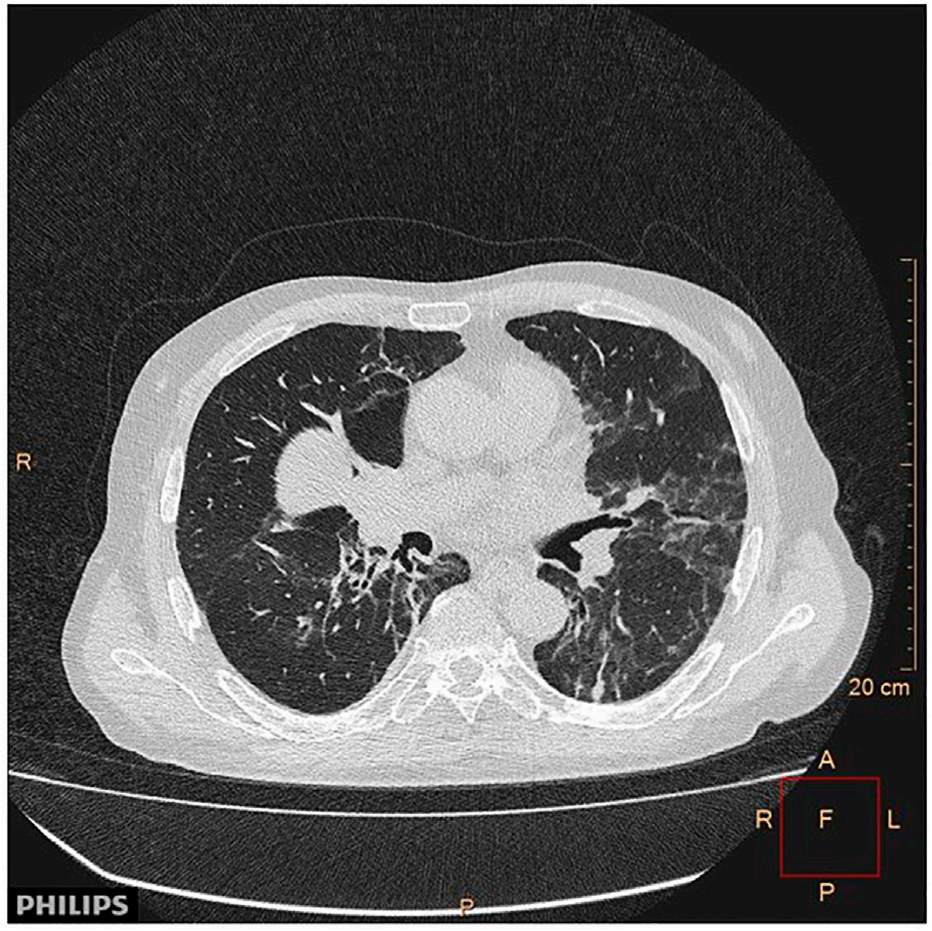
CT image of the pneumonitis caused by everolimus treatment of cancer patient. With permission of Hungarian Society of Clinical Oncology (5).

Most mTOR inhibitor treatment induce pneumonitis (Figure 10). The pathomechanism of this side effect is not yet fully clarified. Taken the role of mTOR pathway in the regulation of the innate and adaptive immune systems into consideration, immune mediated processes probably plays a role in the development of pneumonitis, similarly to the immune checkpoint inhibitors. Interestingly, de frequency of the incidence of pneumonitis varies in different clinical trials, despite it is considered as an „on target” side effect(45). For example, in breast cancer clinical trials the incidence of pneumonitis was found to be 7.6% compared to pancreatic neuroendocrine clinical trials in which the frequency of pneumonitis was much higher, 17% (48, 49). There are also available reports on a much higher incidence, reaching 49% (31). Pneumonitis is a dose dependent adverse event, the severity of pneumonitis regress with dose reduction, and usually ends after discontinuation of mTOR inhibition (45). MTOR inhibitor treatment-induced pneumonitis, similarly to the case of immunotherapy, is associated with treatment outcomes (50). Overall survival (OS) and progression-free survival (PFS) were significantly longer in those individuals, who developed pneumonitis, as compared to those, who did not. Also, stable disease (SD) was more frequent in patients who developed pneumonitis, than in those who did not (45)**.**

#### Dermatological Side Effects, Stomatitis

The mTOR targeted treatments frequently cause a wide range of dermatological and mucosal side effects, such as rash, acne-like lesions and mucositis. The pathomechanism of the dermatological side effects of mTOR targeted therapy is related to the inhibition of EGFR, similarly to EGFR target therapies (45, 46).

#### Hepatotoxicity

The PI3K plays a key role in the survival of hepatocytes, therefore the inhibition of PI3K induces apoptosis and liver injury. Hepatotoxicity is frequently associated with PI3K inhibitor treatment, especially with pan-PI3K and PI3Kδ inhibitor therapy. The hepatotoxicity associated with pan-PI3K inhibitors is usually mild, while the hepatotoxicity induced by the PI3Kδ inhibitor idelalisib, is frequent and severe. Autoimmunity is the possible underlying patomechanism, since the liver biopsy of patient suffering idelalisib induced hepatitis shows activated T cell infiltration and treatment with steroids is usually effective (46).

## Glossary

ARDS: adult respiratory distress syndrome
BF: bisphosphonate
BSP: bone sialoprotein
CML: chronic myelocytic leukemia
CNS: central nervous system
COPD: chronic obstructive pulmonary disease
CSK: cytoskeleton
GFR: growth factor receptor
HgB: hemoglobin
HIF: hypoxia inducible factor
ICI: immune checkpoint inhibitor
IR: insulin receptor
irAE: immune related adverse event
IRS: insulin receptor substrate protein
MLCK: myosin light chain kinase
NET: neuroendocrine tumor
NK: natiral killer
NOS: nitricoxid synthase
NSCLC: non-small cell lung cancer
OBL: osteoblast
OCL: osteoclast
ON: osteonectin
OPN: osteopontin
OS: overall survival
PDGFR: PDGF receptor
PFS: progression-free survival
PTHrP: parathyroid hormon-related protein
RBC: red blood cell
RCC: renal cell cancer
ROS: reactive oxigen species
TK: tirozine kinase
VEGFR: VEGF receptor
